# Malarial Poison

**Published:** 1881-03

**Authors:** W. O’Daniel

**Affiliations:** Bullard’s, Georgia


Medical and Surgical Journal.

VOL. XVIII.] MARCH—1881. [NO. 12.

### Original Communications.

### MALARIAL POISON.

BY W. O'DANIEL, A.M., M.D., OF BULLARD'S, GEORGIA.

We notice in the Louisville *Medical News* of the 16th inst. an extract from Dr. W. C. Maull, of Middleton, Ill., in regard to wire screens to exclude the free passage of poisonous air in malarious districts as a preventive of malarial poison.

We write this article merely to add whatever testimony we may have obtained from actual experience in a practice of fourteen years in a malarious section of country, one in which few enjoy complete immunity from malarial diseases, even with the use of preventive means judiciously and persistently employed.

We have never recommended wire screens, but we are confident they are of much practical utility, but not so efficacious as closed doors and windows after nightfall.

We know of a clerk in a store at Bullard's, Ga., who is surrounded by a low, flat, damp, swampy section of country, where almost every one suffers more or less from fevers of malarial origin during the summer and autumn months. This gentleman consulted me as to the employment of some prophylactic means whereby he might at least mitigate expected attacks and recurrences of intermittent, and bilious fevers. Of course, he expected them, because but few of the inhabitants in that immediate section escaped such fevers.

We advised him to close his doors and windows after sunset, and keep them closed until after sunrise during the "sickly season" as it is called, and that in our opinion he might *at least* enjoy *partial* exemption from bilious remittent and intermittent types of fever.

Our advice was strictly adhered to, and for several years he has been almost free from malarial attacks, while others in the neighborhood, who disregarded the laws of hygiene and prophylaxis in this particular, have been compelled to succumb to repeated attacks of malarial fevers.

In the same neighborhood resides a gentleman, wife and daughter who have been residents of Philadelphia all of their lives (he now being about sixty years old) until about ten years ago, when he concluded to change the monotony of city life for a country residence in Georgia, on the Ocmulgee river.

He having engaged in agricultnral pursuits, was compelled to be out late at night and early in the morning, suffered every year from malarial fevers, enlarged spleen and liver, despite remedial agents, whereas his wife and daughter, who remained indoors between sunset and sunrise, suffered very little from misasmatic fevers. Did time and space admit we could mention many other similar cases.

Now, if the wire screens would as successfully exclude the free passage of the invisible monster who so insidiously insinuates his poisonous influences into our unsuspecting systems, of course they would be far preferable, inasmuch as the ventilation would be so much better, thereby rendering sleeping apartments more comfortable in warm weather than they could possibly be with closed doors and windows, which would successfully preclude the free passage of air, which contains the vitalizing element, oxygen, which is known to exist in all nature, but must necessarily exist in the air we breathe in certain quantities, to insure a healthy condition of the system, yea, even life itself.

Could we not to some extent ventilate our houses when residence in a malarious district is unavoidable by openings of some kind upon their tops, and admit thereby the indispensable element, oxygen, and at the same time exclude malaria, as it always ascends, and never descends? Oxygen would then find its way into our rooms through these openings, or windows, just as it does through windows, stove-pipes, etc.

We believe it is now an acknowledged fact that our systems become saturated with malaria during the night time and damp cloudy weather, when dissipation by the rays of the sun is impossible. At such times we think it advisable for residents of unhealthy locations to keep up constant fires, for the purpose of drying the houses, especially during the summer and autumn months.

The white man long exposed to malarial influences is found to have enlargement of the liver and spleen, while such is rarely ever the case in the black man. A satisfactory reason for the difference I shall not now attempt to give, though we are confident observing and experienced practitioners of medicine in sections of country where malarial diseases prevail, will corroborate this statement.

We do not mean by this that the black man is exempt from malarial influences, but we do not think they are so susceptible as the white man.

Quinine is the great neutralizer of malarial poison, and without it the per cent. of mortality would be much greater than it is in sections where malaria exists. By the daily use of it we are almost enabled to defy miasma. We always use quinine and other preparations of cinchona to break up the paroxysms, and then to prevent a recurrence, which may be looked for on the 7th, 14th and 21st days, we use many tonic preparations. A favorite one with us is:—

℞.—Quinæ sulph. - - - - ʒij

Ferri sulphas, - - - - - ʒiss

Acid arsenitis.

Strychniæ, aa, - - - - gr. ij

Fiat pills, No. lx.

Sig.—Dose, one pill twice or thrice daily after meals.

Of course, the liver and spleen receive their share of attention, and such purgative remedies used as are from time to time indicated.

